# SUMO-1 regulates the conformational dynamics of Thymine-DNA Glycosylase regulatory domain and competes with its DNA binding activity

**DOI:** 10.1186/1471-2091-12-4

**Published:** 2011-02-01

**Authors:** Caroline Smet-Nocca, Jean-Michel Wieruszeski, Hélène Léger, Sebastian Eilebrecht, Arndt Benecke

**Affiliations:** 1 Institut de Recherche Interdisciplinaire, Université de Lille1 - Université de Lille2 - CNRS USR3078, Parc de la Haute Borne - 50 avenue de Halley - 59658 Villeneuve d'Ascq, France; 2 Unité de Glycobiologie Structurale et Fonctionnelle, CNRS UMR8576 - Université de Lille1, Villeneuve d'Ascq, France; 3 Institut des Hautes Études Scientifiques, 35 route de Chartres, 91440 Bures-sur-Yvette, France

## Abstract

**Background:**

The human thymine-DNA glycosylase (TDG) plays a dual role in base excision repair of G:U/T mismatches and in transcription. Regulation of TDG activity by SUMO-1 conjugation was shown to act on both functions. Furthermore, TDG can interact with SUMO-1 in a non-covalent manner.

**Results:**

Using NMR spectroscopy we have determined distinct conformational changes in TDG upon either covalent sumoylation on lysine 330 or intermolecular SUMO-1 binding through a unique SUMO-binding motif (SBM) localized in the C-terminal region of TDG. The non-covalent SUMO-1 binding induces a conformational change of the TDG amino-terminal regulatory domain (RD). Such conformational dynamics do not exist with covalent SUMO-1 attachment and could potentially play a broader role in the regulation of TDG functions for instance during transcription. Both covalent and non-covalent processes activate TDG G:U repair similarly. Surprisingly, despite a dissociation of the SBM/SUMO-1 complex in presence of a DNA substrate, SUMO-1 preserves its ability to stimulate TDG activity indicating that the non-covalent interactions are not directly involved in the regulation of TDG activity. SUMO-1 instead acts, as demonstrated here, indirectly by competing with the regulatory domain of TDG for DNA binding.

**Conclusions:**

SUMO-1 increases the enzymatic turnover of TDG by overcoming the product-inhibition of TDG on apurinic sites. The mechanism involves a competitive DNA binding activity of SUMO-1 towards the regulatory domain of TDG. This mechanism might be a general feature of SUMO-1 regulation of other DNA-bound factors such as transcription regulatory proteins.

## Background

The human Thymine-DNA Glycosylase (TDG) is part of the base-excision DNA repair (BER) machinery targeting G:U and G:T mispairs that did not arise due to replication errors. Indeed, these mismatches frequently occur on double-stranded DNA after spontaneous or catalytically-mediated hydrolysis of cytosine or C^5^-methylated cytosine leading to uracil and thymine, respectively [1-5]. Among the large family of Uracil-DNA Glycosylase enzymes, which initiate BER at G:U lesions, the subclass of TDG proteins exhibits a broader substrate specificity comprising recognition of erroneous thymine bases [6,7]. Many *in vitro *enzymatic studies characterizing the catalysis parameters of TDG-mediated repair on various oligonucleotide substrates [1,8-11] indicate that besides an evolutionary-conserved catalytic domain [12,13] additional N- and C-terminal domains are responsible of this broader specificity of substrate recognition and processing [14-17] with, as a counterpart, a lower enzymatic turnover [10,11,18,19]. A molecular rescue to this poor catalysis efficiency of TDG was found in the SUMO modification of its C-terminus [11] which helps to improve the turnover rate implying a molecular mechanism that competes with product inhibition [11,14,15]. Indeed, the formation of a protruded α-helix within the catalytic domain upon SUMO conjugation was proposed to facilitate the DNA dissociation from the active site [14,15] while the active site of TDG itself remains unchanged upon SUMO-1 conjugation [20]. Furthermore, a conformational change of the TDG N-terminal region, mimicking the deletion of the N-terminus, was proposed to explain the observed improvement of the enzymatic turnover on the G:U glycosylase reaction through a decrease of TDG's binding affinity for its DNA substrates [18,19]. However, the structural and dynamic details of this hypothesis still remain to be established.

The evolutionary-acquired G:T mismatch specificity intriguingly relates TDG to the epigenetic regulation of transcription through DNA methylation at CpG islands [21]. Furthermore, functional interactions with the DNA-methyltransferase Dnmt3a were found to regulate the re-methylation of the newly reconstituted G:C canonical pair after TDG-mediated BER [22]. Recently, TDG and Dnmt3a were found to participate in a pattern of cyclic methylation of the *tff1 *promoter through their respective enzymatic activities [23]. Furthermore, the TDG mismatch repair efficiency was shown to be compromised upon loss of DNA methyltransferase expression and might require a yet unidentified RNA component for full G:T repair activity [24]. TDG acts also as a transcriptional coactivator of nuclear receptor transcription factors like the estrogen and the retinoic acid receptors [25,26], and functionally interacts with other general HAT coactivators like SRC-1 and CBP [27,28]. Again, sumoylation of TDG was found to regulate TDG activity by abolishing interactions with CBP, preventing its CBP-mediated acetylation *in vitro*, and altering the sub-cellular localization of TDG to the PML oncogenic domains [29].

Covalent TDG sumoylation interferes with the intermolecular SUMO-1 binding that is thought to be mediated by two distinct SUMO-binding motifs located at the amino- and carboxy-terminal regions of the TDG catalytic core. The non-covalent SUMO-binding capacity of TDG is also negatively affected by DNA binding through the TDG N-terminal region [29]. It is this non-covalent SUMO-1 binding which stimulates CBP-dependent transcriptional activation [29] and is involved in TDG translocation to PML oncogenic domains, implicating its ability to bind sumoylated PML or other sumoylated proteins found within this nuclear compartment [29,30].

**Figure 1:**
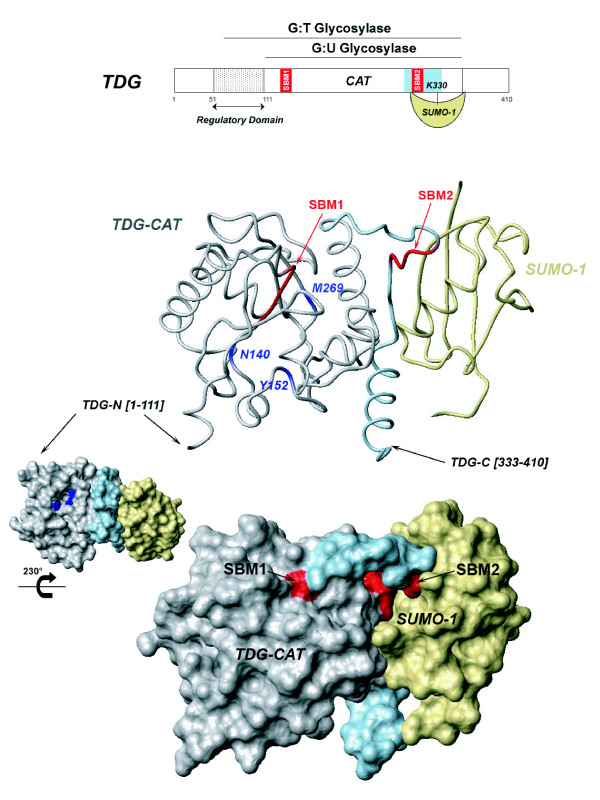
**Schematic representation of TDG domains and the SUMO-conjugation site (upper panel)**. Three-dimensional structure of the SUMO-1-modified TDG-CAT protein (ribbon and surface representations) determined by X-ray diffraction (14) with, indicated between brackets, the junctions of TDG N- and C-terminus. TDG-CAT is represented in grey and the interface with SUMO-1 in blue, SUMO-1 is colored in yellow. The SUMO-binding motifs (SBM1 and SBM2) are indicated in red and the catalytic residues in blue. Molecular models were generated using the Protein Data Bank (PDB) structure (PDB code 1WYW) and processed with the MolMol software (42).

For both SUMO-1 conjugation and intermolecular SUMO-1 binding, the N-terminal domain of TDG was found to be targeted in the modification of TDG function in BER. We have previously reported that the regulatory domain, located in the N-terminus of TDG (see Figure 1), provides an additional non-sequence or mismatch specific DNA binding activity and furthermore established dynamic intramolecular interactions with the core catalytic domain [31]. This interface is altered in the presence of a DNA substrate. Moreover, the conformation of the regulatory domain modulates the TDG glycosylase activity and enzymatic turnover in a mismatch-dependent manner [31]. Here we describe the effects on the conformational dynamics of TDG, and in particular on the regulatory domain, of SUMO-1 conjugation on the one hand and non-covalent SUMO-1 binding on the other. The mechanism of stimulation of TDG glycosylase activity by SUMO-1 is described.

## Results

### SUMO-1 conjugation to TDG affects the C-terminal domain conformation but not the N-terminal region of TDG

The uniformly ^15^N-labeled TDG protein conjugated on lysine 330 to SUMO-1 was produced in E. coli as described [34]. The conjugation site was verified using as a negative control the TDG-K330A mutant under the same conditions for protein production. In this latter control case only the non-modified TDG-K330A protein was isolated after purification as checked by MALDI-TOF MS and denaturing gel electrophoresis (data not shown). Thus sumoylation of TDG under these conditions indeed only occurs on lysine 330.

**Figure 2:**
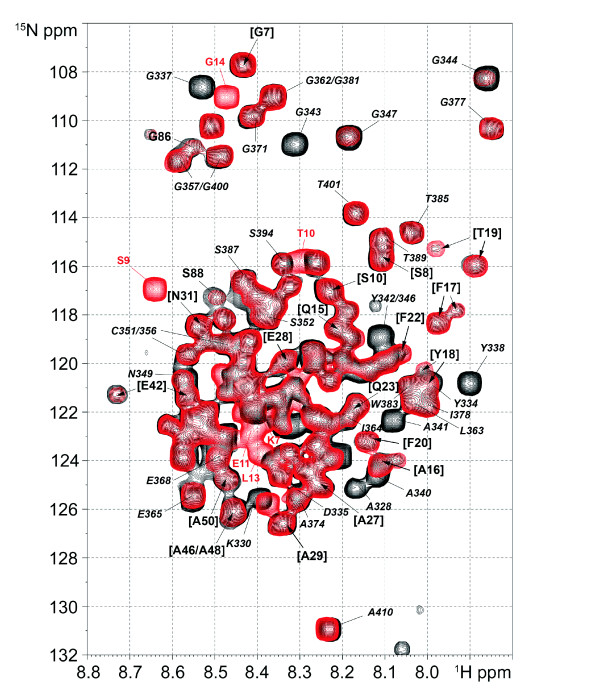
**^15^N-^1^H HSQC spectra of TDG (black) and TDG conjugated to SUMO-1 (red)**. Resonances of the extreme N-terminus of TDG (residues 1 to 50) are annotated between brackets and indicated by arrows, resonances of TDG-RD and TDG C-terminus are in bold and in italic, respectively, and those of SUMO-1 in red.

In our previous NMR study, we have shown that the TDG protein exhibits broad lines on the ^15^N-^1^H HSQC spectrum concerning the large majority of its residues and that only the N- and C-terminus resonances are detectable due to their high degree of flexibility in solution (corresponding to residues 1-50 and 328-410 respectively) [31]. We have also shown critical conformational dynamics for the regulatory domain of the N-terminus (TDG-RD, residues 51-111, see Figure 1). This region, coinciding with a functional domain implicated in specific G:T excision [1], adopts a residual structure in the context of the isolated N-terminus and undergoes a dramatic conformational and dynamic change in the context of the entire protein leading to the disappearance/broadening of corresponding resonances. The disappearance of resonances was shown to be due to intramolecular RD/CAT interactions [31]. As for the unconjugated TDG protein, the acquisition of a ^15^N-^1^H HSQC spectrum on SUMO-modified TDG leads to the detection of random coil regions. Only the 1-50 segment of the N-terminus and the extreme C-terminus display sufficiently sharp resonances (Figure 2). Furthermore, also for SUMO-1, only some N-terminal resonances are observable while the major part of SUMO-1 resonances are too broad to be detected, somewhat mimicking the NMR behavior of TDG-CAT and TDG-RD domains (Figure 2). These data are consistent with the X-ray structure of TDG conjugated to SUMO1 where tight associations between SUMO-1 and TDG-CAT through the C-terminal SBM were highlighted [14]. The resonances of the TDG N-terminal region (residues 1-50) are not perturbed upon SUMO-1-conjugation when compared to non-modified TDG protein. In contrast, the resonances of residues 327 to 347, surrounding the K330 sumoylation site, are significantly broadened (Figure 2), indicating conformational modifications of the TDG C-terminus through covalent sumoylation and no remote perturbations of the N-terminal conformation. We cannot exclude, given the absence of detectable NMR signals that some conformational changes of the TDG regulatory and catalytic domains upon SUMO-1 conjugation occur. Note, however, that based on previous work a structural change of at least the TDG active site after SUMO conjugation is rather unlikely [20].

### TDG/SUMO-1 non-covalent interactions induce conformational changes within the N-terminal regulatory domain and the C-terminal region of TDG

**Figure 3:**
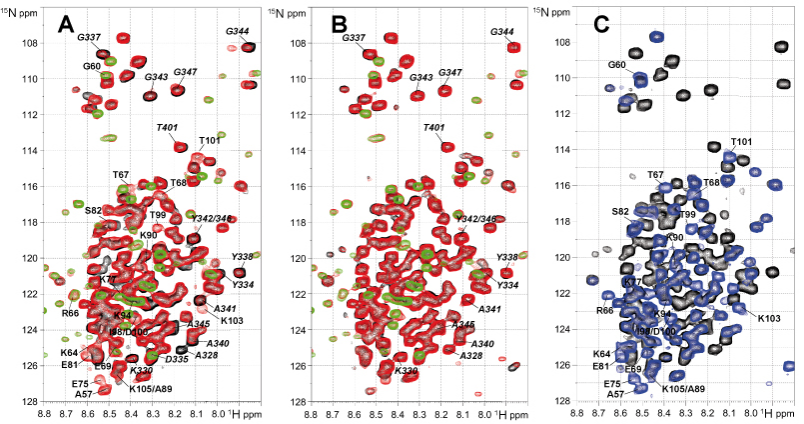
**(A) ^15^N-^1^H HSQC spectra of TDG alone (black) at 20 μM or in presence of 120 μM of SUMO-1 (red)**. Resonances of TDG-RD are annotated in bold and resonances of the C-terminal residues in italic. Some resonances of unlabeled SUMO-1 are detectable at ^15^N natural abundance. (B) ^15^N-^1^H HSQC spectra of ^15^N-TDG-E310Q at 20 μM without SUMO-1 (black) or in presence of 200 μM SUMO-1 (red). The ^15^N-^1^H HSQC of ^15^N-SUMO-1 (green) is shown as a reference to identify SUMO-1 resonances detected at ^15^N natural abundance. Resonances of the C-terminal domain are indicated in italic. Resonances of TDG-RD, indicated in bold, exhibiting a very low intensity on TDG and TDG-E310Q spectra appear with a higher intensity in presence of SUMO-1 for TDG only. (C) The overlay of the ^15^N-^1^H HSQC spectra of the ^15^N-TDG (black) and the isolated ^15^N-TDG-N (blue) identifies the resonances of TDG-RD residues.

It had previously been shown (i) that SUMO-1 can interact with TDG also in a non-covalent manner through apparently two distinct binding sites (residues 133-137 and 308-311, namely SUMO-binding motif SBM1 and SBM2, respectively) located within TDG-CAT (see red shading in Figure 1, as well as the model in Figure 8) [14,29,34] and (ii) that the interactions of TDG with DNA as well as sumoylation of TDG prevent further SUMO-1 intermolecular interactions [29]. The non-covalent interactions with SUMO-1 could be either implicated in the TDG sumoylation process itself - as intermediate states, or in functional interactions between TDG and other sumoylated proteins [29,30]. Moreover, since SUMO conjugation to TDG was shown to reduce its DNA binding activity, which suggests when seen in context of previous works, a putative modification of the TDG N-terminal conformation [11,18,31], we have investigated the intermolecular interactions between TDG and SUMO-1 by NMR spectroscopy. In direct binding experiments, we have not detected chemical shift perturbations of the resonances of the isolated N-terminal domain (residues 1-111) in the presence of a 3-fold excess of SUMO-1 (data not shown). These data confirm that there are no direct interactions between SUMO-1 and the N-terminal domain of TDG. Moreover, in ^15^N-labeled full-length TDG, the resonances of the regulatory domain (residues 51 to 111) become partially detectable upon unlabeled SUMO-1 addition (Figure 3A) while no modification was detected for the first fifty N-terminal residues. We indeed show a number of new resonances on the ^15^N-^1^H HSQC spectrum of the ^15^N-labeled TDG protein in the presence of SUMO-1 (Figure 3A) that match very well with those of TGD-RD observed in the context of the isolated TDG N-terminus (Figure 3C, blue spectrum) indicating that SUMO-1 produces a conformational change of TDG-RD upon binding to SBMs. These resonances are of lower intensity as compared with those of the N[1-42]50]-terminal region suggesting a partial effect on TDG-RD conformation. An increase of RD resonances was measured when adding increasing amounts of SUMO-1 over TDG (ranging from an equimolar amount to a 10-fold excess). We were also able to detect a gradual decrease of signal intensities for some resonances of the TDG C-terminus (from A328 to A345) in presence of SUMO-1 (Figures 3A and see Additional file 1, Figure S1) which indicates a modification of the C-terminal dynamics and conformation upon SUMO-1 intermolecular binding to SBMs. Remarkably, the non-covalent interaction of SUMO-1 and the covalent SUMO-1 modification of TDG induce a perturbation of the same TDG C-terminal resonances. This effect is obviously more pronounced for SUMO-1 conjugation than for the non-covalent binding and leads to the only consistent interpretation that cis and trans SUMO-1 target at least one identical region of TDG-CAT: the C-terminal SUMO-binding motif (SBM2, see Figures 1, 8). To confirm this interaction, we have acquired a ^15^N-^1^H HSQC spectrum on ^15^N-labeled SUMO-1 in presence of TDG. Despite we observed some slight signal perturbations upon TDG addition it seems rather to be induced by weak, non-specific interactions (data not shown). However, an overall 2-fold decrease of SUMO-1 signal intensity in the presence of TDG was noticed with exception of its N-terminal residues (K7-G14) that remain unchanged (see Additional file 2, Figure S2). Hence, the SUMO-1 population bound to TDG cannot be detected on the ^15^N-^1^H HSQC spectrum of ^15^N-labeled SUMO-1 as already observed for SUMO-1 conjugated to TDG. Only the remaining free SUMO-1 molecules are detected. Taken together, our data indicate that non-covalent interactions between SUMO-1 and TDG exist, but do not directly involve the TDG N-terminus which is in accordance with previous studies [29,30].

### SUMO-1 does not interact with TDG-E310Q

Having observed the importance of at least the C-terminal SBM also in the case of covalent sumoylation of TDG, we decided to further analyze the SUMO-1 interaction sites within TDG-CAT. Since two SUMO-binding motifs had been previously proposed, one at the amino- and another at the carboxy-terminal part of TDG-CAT [29], we wanted to determine which SBM mediates the N- and/or C-terminal conformational changes which we were able to detect by NMR. We have produced three SBM mutants by either mutating the SBM1 (D133A) or SBM2 (E310Q) or both (D133A/E310Q) similarly to Mohan and co-workers [29]. The ^15^N-labeled proteins were initially analyzed by NMR and circular dichroism spectroscopy (see Additional file 3, Figure S3). Our data show that the D133A mutation of the conserved DIVII SUMO recognition sequence of the amino-terminal SBM (SBM1, Figure 1) leads to a significant misfolding of the protein and consequent aggregation (see Supporting Information, Figures S3A and S3C) and thus cannot be considered for further interaction studies with SUMO-1. Such a misfolding could be assigned to the experimental conditions (protein concentrations, absence of glycerol) or heterologous protein overexpression in *E. coli *but it is not observed, however, for wild-type TDG or the TDG-E310Q mutant (as described in the following paragraph) that are produced and investigated under the same conditions. It should also be noticed that the IVII motif, with exception of the D133 residue, is not solvent-accessible in both the non- and SUMO-modified TDG-CAT structures (Figure 1) [14,20]. While the D133A mutation indeed might lead to loss of SUMO-1 binding as described in [29], our data raise the possibility that loss of interaction could also be the result of a more general, unspecific effect of TDG misfolding in this part of the molecule and subsequent aggregation of TDG-D133A into high-molecular weight precipitates.

In contrast, the TDG-E310Q mutant behaves as the TDG wild-type protein and few discrepancies were detectable in far-UV spectra obtained by circular dichroism (see Additional file 3, Figure S3C) as well as on the HSQC resonances between both spectra (see Additional file 3, Figure S3B). This is, given our previous analysis of TDG-CAT NMR behavior [31], explained by the fact that the mutated residue is part of the very rigid region not detected in the HSQC spectra. Moreover, since few differences between mutant and wild type proteins are observed when comparing the HSQC spectra, we can reasonably assume that the E310Q mutation does not, unlike the D133A mutation, strongly affect the structure of TDG.

We have further investigated the SUMO-1 binding to TDG-E310Q. Under the same conditions used as for wild-type TDG, no modification of neither C-terminal nor RD resonances of TDG-E310Q were detected in the presence of a 10-fold molar excess of SUMO-1 (Figure 3B) indicating that (i) SUMO-1 binding to TDG is abolished by the E310Q mutation and (ii) SUMO-1 binding to the TDG C-terminal SBM is solely responsible for both the C- and N-terminal conformational changes. Moreover, in contrast to wild-type TDG, the overall signal intensity of ^15^N-SUMO-1 does not decrease in presence of a 3-fold excess of TDG-E310Q (data not shown), confirming that SUMO-1 does not interact with TDG-E310Q. Furthermore, the CD spectra of TDG or TDG-E310Q in presence of SUMO-1 point to a slight modification of protein structures for the wild-type TDG only confirming the TDG/SUMO-1 intermolecular interaction and subsequent structural rearrangement (Additional file 4, Figure S4).

### No competition between cis and trans SUMO-1 for TDG-CAT binding

Interestingly, SUMO-1 was also able to bind SBM2 in the context of sumoylated TDG (see Additional file 5, Figure S5A). We have detected modifications of the C-terminal resonances of ^15^N-labeled sumoylated TDG when adding a 10-fold molar excess of unlabeled SUMO-1 as well as appearance of TDG-RD resonances similarly to unmodified TDG. However, except of SUMO-1 resonances observable at natural abundance, no additional ^15^N-labeled SUMO-1 signals coming from sumoylated TDG were detected indicating that SBM2 bound SUMO-1 does not displace intramolecular SUMO-1. These data show that intermolecular SUMO-1 binding does not fully compete with cis SUMO-1 and that SBM2 remains accessible to SUMO-1 interactions. Based on these observations, we can speculate for a larger C-terminal SBM than the one that has been described [28]. Additionally, the ^15^N-^1^H HSQC spectrum of the sumoylated TDG-E310Q mutant shows no significant modification of TDG-E310Q resonances and no SUMO signals except the amino-terminal residues also detectable for the SUMO-modified wild-type TDG (see Additional file 5, Figure S5B). These data confirm the existence of distinct SUMO interfaces for either cis or trans SUMO-1 moieties.

Taking together the structure of the SUMO-1 modified TDG-CAT protein and our NMR data, the SUMO-1 conjugation rather acts on the TDG C-terminal conformation with no or little impact on the TDG-RD conformation. In contrast, the SUMO-1 non-covalent binding to the C-terminal SBM is able to structurally modify both the N- and C-terminal regions of TDG and sumoylated TDG. Based on the observations reported here, we conclude that SUMO-1 does not adopt the same orientation as in the sumoylated protein. Interestingly, SUMO-1 non-covalent binding leads to a partial RD displacement from its CAT interface indicating an effect of steric hindrance rather than overlapping binding interfaces on the CAT domain which is in good agreement with our previous suggestion for the putative localization of the RD interface on the CAT domain [31].

### SUMO-1 does not interact with the C-terminal SBM in presence of DNA

**Figure 4:**
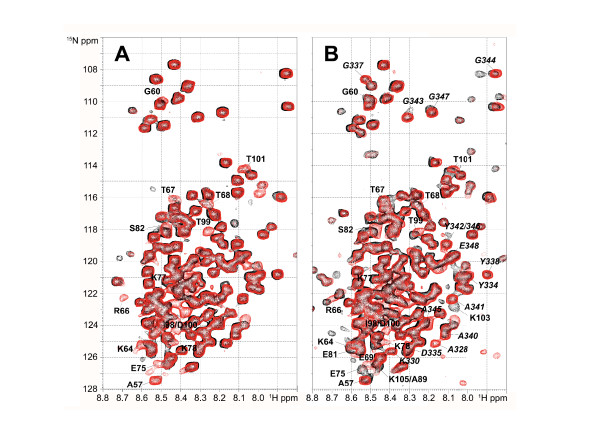
**SUMO-1 influences the TDG-DNA interaction**. (A) ^15^N-^1^H HSQC spectra of TDG at 20 μM without dsDNA substrate (black) or in presence of 50 μM of a 37-mer dsDNA substrate containing a G:T mismatch (red). (B) ^15^N-^1^H HSQC spectra of TDG in presence of 120 μM SUMO-1 without DNA (black) or in presence of 50 μM of DNA substrate containing a G:T mismatch (red). Resonances of TDG-RD residues are annotated in bold and resonances of C-terminal residues in italic.

It has been shown that SUMO-1 intermolecular binding is strongly reduced by TDG's association with DNA [29]. Given our previous results concerning TDG-RD/DNA interactions [31], we have examined the effect of DNA heteroduplexes containing a G:U or a G:T mismatch on TDG conformation in the presence of SUMO-1. Some weak additional resonances matching with those of the isolated TDG N-terminus bound to DNA heteroduplexes are observed on the ^15^N-labeled TDG HSQC spectrum (Figure 4A and Additional file 6, Figure S6) suggesting that DNA substrates containing either a normal G:C pair or a G:T/U mismatch (in 2.5-fold excess) can displace similarly TDG-RD from its TDG-CAT interacting surface (see Additional file 6, Figure S6). Furthermore, no signal perturbation of TDG-RD or A328-A345 region was observed upon SUMO-1 addition (Figure 4B). These data indicate that a DNA heteroduplex containing either a G:U or a G:T mismatch induces a conformational modification of TDG-RD, this effect being independent of SUMO-1 being present or not, and prevents SUMO-1 binding to the C-terminal SBM which is in accordance with previous works [29]. DNA binding to TDG-CAT likely modifies the SBM2 conformation or accessibility so that it prevents any SUMO-1 interactions. We can not exclude that SUMO-1 could modify the binding affinity of TDG to DNA as it has been shown previously in an indirect manner [29]. However, given the dissociation constant of the TDG/DNA complex (in the nM range) and the relatively high protein concentrations that must be used for NMR studies (in the range of at least 10 μM), the SUMO-induced decrease of TDG/DNA affinity (leading to a shift or a decrease of RD resonances) is not strong enough to be detected since, with a 20 μM sample, TDG, and more particularly the RD, is still saturated with DNA whether SUMO is present or not.

### SUMO-1 stimulates the glycosylase activity of TDG and TDG-E310Q

**Figure 5:**
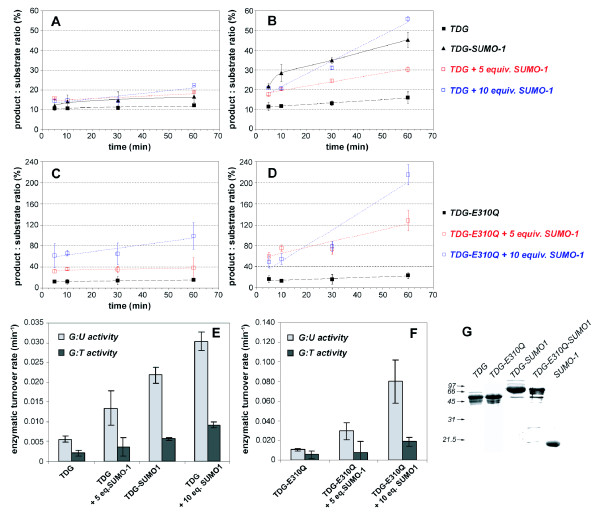
**Glycosylase kinetics of TDG proteins on G:T (A) and G:U (B) repair**. The curve of unmodified TDG (black square) is represented as broken lines for reference. The kinetics curves of sumoylated TDG (black square) and TDG-ΔN (black diamond) are represented by black lines. The kinetic curves of TDG in presence of 5 equivalents (red square) and 10 equivalents (blue square) of SUMO-1 are represented by dotted lines. (C, D) As in (A, B) using TDG-E310Q protein. (E, F) Graphical representation of the enzymatic turnover rates for the G:T (dark grey bars) and G:U (light grey bars) glycosylase activity of TDG (E) and TDG-E310Q (F). (G) Control of protein integrity in SDS-PAGE.

**Figure 6:**
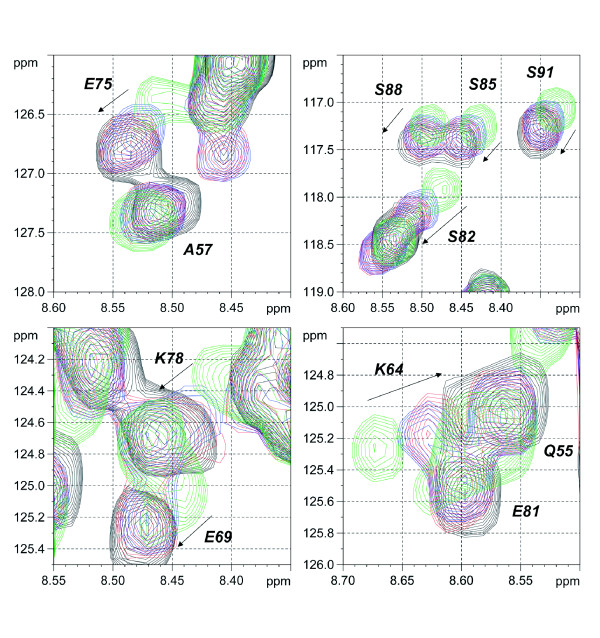
**Glycosylase kinetics of TDG and sumoylated TDG proteins in absence and presence of free SUMO-1 or BSA on G:U and G:T repair in NMR buffer**. (A, B) G:U repair activity was measured for TDG (A) or sumoylated TDG (B) in the presence or absence of five equimolar amounts of free SUMO-1 or BSA at pH 6.6. (C) G:T repair activity of sumoylated TDG in the presence or absence of five equimolar amounts of free SUMO-1 or BSA at pH 6.6.

**Figure 7:**
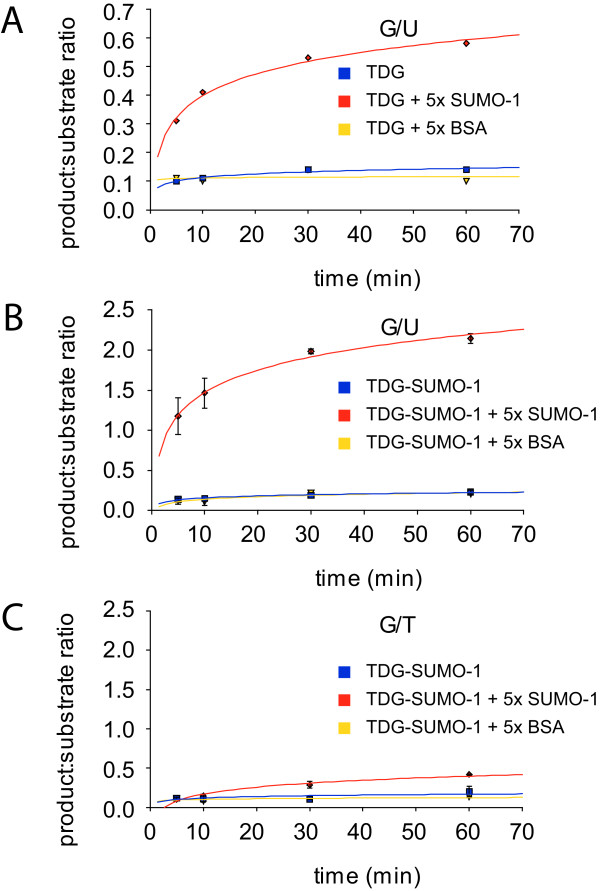
**Competition between TDG-RD and SUMO-1 for DNA binding**. 20 μM ^15^N-labeled TDG-N with an equimolar amount of a dsDNA-25mer substrate containing a G:T mismatch (blue spectrum) was submitted to a 4-fold molar excess of SUMO-1 (80 μM) (red spectrum). Spectra of isolated ^15^N-TDG-N at 20 μM alone (black spectrum) and in presence of 80 μM of the same DNA substrate (green spectrum) were given as references. A significant displacement of RD/DNA complex by SUMO-1 is observed for residues E75, K78, S82, S85, S88 and S91. Absence of competition is found for residues Q55, A57, K64 and E69 indicating a partial competition between SUMO-1 and TDG-RD centred on the region 75 to 91.

Although intermolecular SUMO-1 binding did not occur in presence of DNA or with the C-terminal SBM mutation, we have observed a stimulation of the glycosylase activity of wild-type and E310Q mutant TDG proteins. Using a glycosylase assay, we have measured a slight increase of TDG and TDG-E310Q activities and turnover rates upon sumoylation or SUMO-1 addition on the G:T glycosylase reaction (Figure 5A, C). In contrast, the G:U activities and enzymatic turnovers were very sensitive to sumoylation (for wild-type TDG) or SUMO-1 addition in a dose-dependent manner (Figure 5B, D). We have measured a G:U turnover rate increased by a factor of 3.9 for the sumoylated TDG as compared to the non-modified TDG, while a 2.4- and 5.4-fold increase was observed upon addition of 5 and 10 molar equivalents of SUMO-1, respectively (Figure 5C). We have shown in control experiments that the non-covalent SUMO-1 effect is highly specific as same amounts of BSA did not induce such a stimulation of TDG and sumoylated TDG glycosylase activities (Figure 6). Furthermore, indeed, free SUMO-1 can also further increase G:T and G:U processivity of sumoylated TDG unlike BSA (Figure 6). Finally, the increase in activity of TDG that we postulated based on NMR experiments can be shown to take place under the same experimental conditions as the protein-protein and protein-DNA interactions, that is in NMR buffer at pH 6.6 (Figure 6). Note that while TDG's processivity drops by almost an order of magnitude when using acidic buffers, however, the specific stimulation by sumoylation and free SUMO-1 is clearly detectable and comparable to the one detected under standard experimental conditions (Figure 5, Figure 6, and data not shown). Hence SUMO-1, similarly to the sumoylation of TDG, positively acts on the G:U glycosylase activity and also improves albeit weakly the G:T activity. Hence, despite a disruption of SBM2/SUMO-1 interactions in presence of DNA or upon SBM2 mutation, SUMO-1 was still able to activate TDG glycosylase activities on both G:T and G:U substrates in a dose-dependent manner suggesting an indirect mechanism where the TDG/SUMO-1 interaction is not directly responsible for the up-regulation of glycosylase activity (see also Figure 7, and Discussion section).

### SUMO-1 competes with TDG-RD for DNA binding

Since SUMO-1 does not interact with the TDG C-terminal SBM upon SBM mutation or DNA addition, it rather seems that SUMO-1 acts indirectly on TDG activity by an unknown mechanism. We have thus investigated the ability of SUMO-1 to directly interact with DNA and shown a non-specific but detectable interaction using NMR spectroscopy and gel shift assays [34]. In this study, we have also demonstrated competition between SUMO-1 and TDG-RD for DNA binding with EMSA.

Here, we demonstrate the ability of SUMO-1 to displace RD from DNA in a direct competition experiment using NMR methodology. In presence of an equimolar amount of a double-stranded 25-mer DNA substrate containing a G:T mismatch, some weak chemical shift perturbations of TDG-RD were observed and are more pronounced with a 4-fold molar excess of the same substrate (Figure 7). Adding a 4-fold molar excess of SUMO-1 to the equimolar TDG-N: DNA mixture induces a shift of RD resonances towards those for the free RD. This effect concerns resonances for residues comprised in the region from position 75 to 91, indicating a partial competition of SUMO-1 with the RD for DNA binding (Figure 7). For the N- and C-terminal parts of TDG-RD, no competition was observed. Since the TDG-RD has a weak, non-sequence specific DNA binding activity [31,34] that contributes to reinforce TDG binding to DNA at the expense of the enzymatic turnover, a partial competition between SUMO-1 and TDG-RD could therefore sufficiently destabilize the TDG/DNA complex with, as a consequence, an increase of G:T/U turnover. Given the relatively low affinity of TDG-N for DNA (estimated to 100 - 500 μM), a substantial amount of free DNA (more than 85%) is found within the equimolar TDG-N: DNA mixture possibly leading to many unproductive (in terms of competition) SUMO-1: DNA complexes. In the context of the entire TDG, as the presence of a SBM will favor the recruitment of SUMO-1 leading to a significant increase of its local concentration in the near vicinity of RD, the competition between SUMO-1 and RD might be more pronounced. We have shown that such a competitive mechanism is indeed feasible [34].

## Discussion

**Figure 8:**
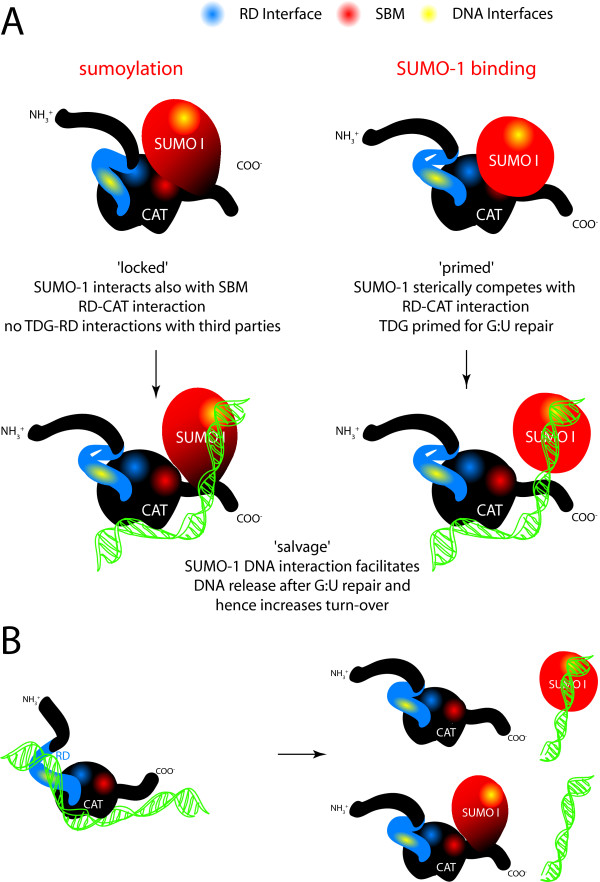
**A schematic representation of the main results obtained**. (A) SUMO-1 covalent conjugation to K330 leads to a change in the C-terminal conformation of TDG. SUMO-1 thereby also interacts with SBM2. TDG-RD is not displaced from the TDG-CAT domain and hence can rest in its "closed" conformation. Sumoylation thereby influences third party interactions with the RD and therefore "locks" the RD. The presence of free SUMO-1, just as covalent SUMO-1 addition to "open" TDG conformers, increases especially G:U turnover rates ("primed"). Note that also SUMO-modified proteins might be recruited to TDG via SBM2 and have similar effects on TDG's turnover rate. SUMO, when bound via SBM2, sterically competes with TDG-RD for the TDG-CAT surface. The TDG-RD hence adopts a partially "open" conformation which leads to increased G:U repair activity. Also, when SUMO is bound to the SBM2 site, the C-terminus of TDG adopts a conformation similar to the one in the sumoylated protein. The enzymatic turnover especially on G:U mismatches is enhanced through the DNA interaction of either SBM2 recruited or covalently attached SUMO-1. Note that the effect in the case of transient SBM2 interaction is likely due to a local concentration effect as it does not require prolonged SBM2 binding by SUMO. (B) SUMO-1 conjugation or binding to the SBM2 might also occur post-repair once TDG has been trapped on its abasic G:- product to salvage TDG activity by overcoming product inhibition. In the case of non-covalently bound SUMO-1 alternatively a third protein carrying the SUMO-1 group might bring SUMO-1 sufficiently close to TDG for the 'salvage' effect.

We have found that the posttranslational modification of TDG by SUMO-1 (i) has no detectable effect on the conformational dynamics of the regulatory domain and rather acts on the TDG-CAT [14] and TDG C-terminal conformations (Figure 2) and (ii) stimulates both G:T and G:U glycosylase activities with a more pronounced effect on G:U substrates (Figure 5). It has been shown that SUMO-1 covalent attachment to TDG results in a destabilization of the TDG/DNA complex leading to increased TDG turnover [14]. It has been proposed that SUMO-1 conjugation by mimicking the effect of N-terminal domain truncation on the TDG glycosylase turnover rates could induce long-range conformational changes on this TDG N-terminal domain [18,19]. However, no modification of the N-terminal conformation was detected on full-length TDG conjugated to SUMO-1 by NMR spectroscopy. In contrast, the SUMO-1 non-covalent interaction through a unique SBM localized at the C-terminal region of TDG-CAT (SBM2) competes with the TDG regulatory domain for the binding to the catalytic domain. SUMO-1 thereby is able to partially displace the regulatory domain from the RD/CAT interface leading to a "primed" extended conformation of TDG-RD (Figures 3 and 8) which preserves a sequence-independent DNA binding activity (Figures 4 and 8) as previously observed [31]. Furthermore, since a modification of the C-terminus conformation has been observed resembling the effect of covalent SUMO-1 modification (Figure 3A), it was possible to show that the intermolecular binding of SUMO-1 induces the same modification of the TDG-CAT structure. Moreover, we have demonstrated that both N- and C-terminal conformational modifications were only induced by SUMO-1 binding to the C-terminal SBM (SBM2 in Figures 1, Figure 4) and intermolecular SUMO-1 binding still occur in the context of sumoylated TDG.

Similarly to a DNA substrate containing a normal G:C pair [31], DNA containing a G:T/U mismatch alters the RD/CAT interface and stabilizes the RD extended conformer (see Additional file 6, Figure S6). The RD in its extended conformation interacts with DNA in a sequence-independent manner. Such interactions preserve the RD/DNA contacts essential for the G:T processing while the RD/CAT interactions contributes to decrease the G:T/U turnover rates [31]. Remarkably, SUMO-1 does not modify the RD conformational equilibrium in the presence of DNA and apparently does not interact with TDG in presence of DNA (Figure 4). However, SUMO-1 stimulates the TDG glycosylase activity in a concentration-dependent manner on both G:T and G:U mismatches. Also, with the TDG-E310Q SBM2 mutant, the stimulation effect of SUMO-1 on TDG-E310Q activity can still be observed for G:T/U substrates (Figure 5). While our data show that the SBM1 motif is highly unlikely to be functional for SUMO binding due to it being buried inside the hydrophobic core of the CAT domain, and given the absence of any chemical shift perturbations in NMR experiments using TDG-E310Q in the presence of SUMO, we demonstrate that the effect on the BER activity of TDG is independent of SUMO binding to TDG. It is likely that SUMO-1 facilitates the TDG/DNA dissociation by competing with TDG-RD for DNA binding, as we have shown weak, but significant non-sequence specific interactions of SUMO-1 with DNA duplexes. Indeed, the molecular contacts of TDG-RD with DNA stabilize the TDG/DNA complex leading to a tight association of DNA and a poor turnover rate [10,18]. SUMO-1 by competing with TDG-RD for DNA binding would destabilize the TDG/DNA complex and thus salvage TDG activity (Figure 8). The RD/SUMO-1 competition has little incidence on the G:T excision but significantly increases the G:U activity and turnover rate in a SUMO-1 concentration-dependent manner, thereby mimicking SUMO-1 conjugation. Interestingly, SUMO conjugation was already found to negatively regulate the DNA binding activity of the transcription factor HSF2 in a way that could resemble the non-specific binding we describe here [35]. In the binding experiments we have performed, a large excess of free SUMO-1 was used in order to compete with either the intramolecular SUMO-1 in the sumoylated proteins or the TDG-RD, which is by nature covalently bound to TDG-CAT. In both cases, we have to take into account the concentration effect of SUMO-1 or TDG-RD due to covalent attachment. To compete with such high local concentrations, a significant excess of free SUMO-1 has to be employed in the competition or BER experiments. Note however that in our experiments quantitatively SUMO-1 modified proteins were used which does not necessarily reflect the situation in the cell where low levels of sumoylation that are detected within the cell (usually less than 5%) [36]. Therefore, very distinct effects should be observed with free SUMO-1 on the one hand and covalently attached SUMO-1 on the other.

Interestingly, whether the sumoylation of TDG, its intermolecular interaction with SUMO-1 or both is implicated in the regulation of its function *in vivo *is still not clear. SUMO-mediated interactions of TDG with SUMO-modified proteins could also modulate TDG activity on DNA repair, in a manner similar to the sumoylation of TDG itself. It has been shown that SUMO-1 binding activity of TDG is essential for CBP activation and localization to Promyelocytic leukemia protein Oncogenic Domains [29]. In contrast with the SUMO-1 conjugation, the non-covalent SUMO-1 binding can act in a concentration-dependent manner and would be a more flexible way to regulate TDG glycosylase activity in a sense that it does not require the recruitment of the sumoylation (E1, E2, E3 enzymes) and de-sumoylation machinery [36]. SUMO-1 concentrations in a particular nuclear compartment be it free or conjugated to another protein, could hence result in fine tuning of TDG functions, similar to mechanisms proposed for other sumoylated or SUMO-1 binding proteins [37-39]. It has been proposed that, due to small protein-protein interfaces between SUMO-1 and SBM, this interaction falls within the high micromolar range. High affinities could further result from binding to a sumoylated protein through both a SBM and a second low-affinity interaction site [29,34].

Furthermore, SUMO-1 intermolecular binding could have another function like modifying the TDG interface for its cellular partners, more particularly the RD accessibility, as already described for SUMO conjugation to a transcription factor [37] not for SUMO non-covalent binding. A number of studies have pointed to a central role of the RD in mediating protein-protein interactions [22,25,26,28]. A SUMO-induced conformational change of the RD therefore implies a modification of the molecular interactions not only between the latter and TDG's substrates but also its interaction partners (Figure 8A). Among them is the CREB binding protein (CBP), which could be a target of the SUMO-induced RD conformational changes. Indeed, CBP is sumoylated on three lysine residues located in a region close to the HAT domain and mediates acetylation at four positions within the RD through its acetyltransferase activity. A dual interacting surface, SBM/SUMO-1 on one hand and RD/HAT on the other, leading to a high affinity complex, would involve the SUMO-1 activity of TDG not only for interaction with sumoylated CBP but in modifying the TDG-RD structure in a conformation more favorable to CBP interaction and subsequent acetylation. Consistent with this, the stimulation of CBP-mediated transcription by SUMO-1 binding indicates a possible role of the RD conformational dynamics in the regulation of TDG/CBP interactions [29]. It would be now interesting to investigate at the molecular level whether the RD conformational changes we have observed with free SUMO-1 are reproducible with a sumoylated protein and whether this SUMO-1 binding activity stimulates the interaction.

Finally, a model in which sumoylation or SUMO-1 binding to TDG occurs only once TDG has performed the glycosylase reaction and remains, due to the poor product dissociation rate, trapped on the abasic G:- site would also be consistent with all the experimental evidence available today. In this case sumoylation or SUMO-1 interactions would indeed constitute a salvage pathway removing TDG from lesions in order to allow repair to proceed. Such a mechanism might also explain why SUMO conjugating enzymes seem systematically associated with different DNA repair complexes [38].

## Conclusions

SUMO-1 increases the enzymatic turnover of TDG by overcoming the product-inhibition of TDG on apurinic sites. The mechanism involves a competitive DNA binding activity of SUMO-1 towards the regulatory domain of TDG. This mechanism might be a general feature of SUMO-1 regulation of other DNA-bound factors such as transcription regulatory proteins. The fact that SUMO-1 can interact with DNA in a non-sequence specific manner has broader implications for the role of SUMO in DNA repair and transcription regulation. Several so-far intriguing observations of SUMO activity in both processes [35-38] might find similar explanations of DNA binding competition or allosteric regulation through SUMO-modified DNA interaction properties.

## Methods

### Plasmids

Full-length TDG (residues 1-410) and its isolated N-terminal domain (residues 1-111) were cloned in the pGEX-6P-1 plasmid (GE Healthcare) into the BamHI/EcoRI cloning sites. Oligonucleotide primers used to generate TDG and TDG-N fragments by PCR were as follows: 5'-GATCGGATCCATGGAAGCGGAGAACGCGGGC-3' as the forward primer and 5'-GATCGAATTCTCAAGCATGGCTTTCTTCTTCCTG-3' or 5'-GATCGAATTC TCAAAAACGGTCTACTTTTCTTTTTAC-3' as the reverse primer for TDG and TDG-N, respectively. TDG mutants were produced by site-directed mutagenesis according to the experimental procedures described in [41]. One single (D133A within the SBM1 or E310Q within the SBM2) or two mutations (D133A/E310Q) were generated using this method. pGEX-6P-1 plasmid containing the wild type TDG nucleotide sequence served as a template for mutagenesis. Oligonucleotide primers used to generate the individual mutations were as follows: TDG-D133A: 5'- GACCTTCAATCTGGcCATTGTCATTATTGGCATAAAC CCG-3'; TDG-E310Q: 5'-CGAAATATGGACGTTCAAcAGGTGCAATATACATTTG ACC-3' (the mutated codons are indicated by underlined sequences and the mutated bases by minor characters).

### Expression and purification of recombinant TDG, TDG SBM mutants, SUMO-1 and SUMO-conjugated TDG

Full-length TDG (residues 1-410), its isolated N-terminal domain (residues 1-111) and SUMO-1 proteins were overexpressed in BL21(DE3) strain as GST fusion proteins. Bacteria were grown at 37°C in M9 minimal medium reconstituted with 2 g/l glucose, 1 g/l ^15^N-labeled ammonium chloride, 1 mM MgSO_4_, MEM vitamin cocktail (Sigma) (or in LB medium for the production of unlabeled proteins) and 100 mg/l ampicilline. Protein expression was induced overnight at 20°C following 0.5 mM IPTG addition. Cells were harvested and resuspended in extraction buffer (PBS, 10% glycerol, 1% Triton X-100, 10 mM EDTA, 2 mM DTT) complemented with a protease inhibitor cocktail (Complete, Roche). Cell lysates were obtained by incubation of 0.25 mg/ml lysozyme with the cell suspension in extraction buffer complemented with RNase and DNase followed by brief sonication steps. The soluble extract was isolated by centrifugation. GST-fusion proteins were purified on a Glutathione Sepharose resin (GE Healthcare). Soluble extracts were incubated for 3 hours at 4°C with 25 to 100 μl resin per milliliter of soluble extracts. Unbound proteins were extensively washed away with a GST wash buffer (PBS, 5% glycerol, 1% Triton X-100, 10 mM EDTA) and TDG proteins were eluted by digestion with Precission Protease using 25 μg/ml of resin (GE Healthcare) in one bead volume of elution buffer (50 mM Tris-Cl pH 8.0, 150 mM NaCl, 2% glycerol, 0.1% NP-40, 10 mM EDTA, 5 mM DTT). The reaction was allowed to proceed at 4°C for 20 hours. Then beads were eluted twice with one bead volume of elution buffer. GST-SUMO-1 was eluted in one bead volume of elution buffer (50 mM Tris-Cl pH 8.0, 200 mM NaCl, 5 mM EDTA, 5 mM DTT) containing 10 mM of reduced (L)-glutathione and SUMO-1 was obtained by an overnight incubation with 1 unit of thrombin per mg of protein at room temperature. Proteins were concentrated and purified by gel filtration on a preparative Superdex75 column (GE Healthcare) equilibrated in NMR sample buffer. Proteins were concentrated to obtain final concentrations of 100 μM for TDG proteins or 500 μM for SUMO-1. The protein homogeneities were verified on denaturing polyacrylamide gel, the molecular mass and isotopic labeling by MALDI-TOF mass spectrometry.

TDG-SUMO1 was produced by co-transforming the BL21(DE3) strain carrying the pGEX-6P-1-hTDG plasmid with the pT-E1/E2/SUMO1 vector (a kind gift from H. Saitoh) [32]. Selection of BL21 colonies carrying both plasmids was performed by ampicilline/chloramphenicol double-selection as described [32]. Unlabeled TDG-SUMO1 was produced in LB medium and ^15^N-labeled TDG-SUMO1 in M9 minimal medium as previously described for TDG with 2.5 g ^15^N-labeled ammonium chloride as nitrogen source [34]. The induction phase was performed overnight at 25°C with 0.2 mM IPTG. The purification was realized as described for TDG with an additional intermediary purification step of cation exchange chromatography on HiTrap SP column (GE Healthcare). The column was equilibrated in 50 mM NaiPO_4 _pH 7.4, 10% glycerol, 1 mM DTT (buffer A) containing 10 mM NaCl and TDG-SUMO-1 protein was eluted at a flow rate of 2 mL/min with a linear gradient of NaCl from 0 to 100% buffer B (buffer A with 500 mM NaCl) in 5 column volumes.

TDG mutants were expressed and purified following the same procedure as the wild type TDG protein. Expression profiles were comparable to wild-type protein, but the protein quantities obtained for TDG-D133A and TDG-D133A/E310Q after the first purification step were significantly lower than for TDG wild-type and TDG-E310Q proteins.

### Protein-protein interactions between TDG, TDG-E310Q or SUMO-conjugated TDG and SUMO-1 monitored by NMR spectroscopy

NMR experiments were performed at 293 K on a Bruker DMX 600 MHz spectrometer (Bruker, Karlsruhe, Germany) equipped with a cryogenic triple resonance probe head. All ^1^H spectra were calibrated with 1 mM sodium 3-trimethylsilyl-d(3,3',2,2')-propionate as a reference. All ^1^H-^15^N HSQC spectra were recorded in an aqueous buffer composed of: 100 mM NaiPO_4 _pH 6.6, 1 mM EDTA, 1 mM DTT, 5% D_2_O. ^1^H-^15^N HSQC spectra were recorded on 20 μM-samples of ^15^N-labeled proteins with at least 256 scans per increment and 128 dummy scans, 128 points in the nitrogen dimension and 1024 points in the proton dimension.

Direct binding studies were performed by NMR spectroscopy on (i) the ^15^N-labeled isolated TDG N-terminus (residues 1-111) at 20 μM and a 3-fold excess of unlabeled SUMO-1, (ii) the ^15^N-labeled TDG at 20 μM in presence of a 1-, 3-, 6-, or 10-fold excess of unlabeled SUMO-1 and, conversely, (iii) ^15^N-labeled SUMO-1 at 30 μM in presence of a 3-fold excess of unlabeled TDG or TDG-E310Q. The ^15^N-labeled TDG-E310Q mutant and SUMO-modified TDG was analyzed at 20 μM in presence of 10 equivalents SUMO-1.

### Interactions of TDG, TDG-N and SUMO-1 with G:T/U-containing dsDNA

Annealing of oligonucleotides was performed by heating 1 mM solutions for 5 min at 100°C and cooling down the mixtures slowly to room temperature to obtain double-stranded 37-mers containing G:T or G:U mispairs. These solutions were lyophilized and dissolved at 50 μM final concentration in a 20 μM-solution of ^15^N-labeled TDG in a buffer constituted by 100 mM NaiPO_4 _pH 6.6, 1 mM DTT and 1 mM EDTA. The SUMO-1 binding activity of TDG was investigated on a 20 μM-solution of ^15^N-TDG in presence of a 2.5-fold excess of G:T or G:U mismatch-containing 37-mers with addition of 5 molar equivalents (100 μM) of unlabeled SUMO-1. ^15^N-^1^H HSQC spectra were acquired for each condition with ^1^H and ^15^N spectral windows of 16 and 36.5 ppm, respectively, and with 256 scans. Interactions of SUMO-1 with DNA was performed with a 25-mer double-stranded DNA substrate containing a G:U mismatch under the same conditions described above with 20 μM of ^15^N-labeled SUMO-1 and 135 μM DNA. For the DNA substrates, the sequences of the 5'-3' strands are GAATTCGATAGGTTCCACGGGTACTCGAAGCGGATCC and GATAGGTTCCAC GGGTACTCGAAGC for the 37- and the 25-mer, respectively with, underlined, the base involved in the G:T/U mismatches. The chemical shift perturbations of individual resonances were calculated using the following Eq 1.

(1) $$\Delta \delta \left(\text{ppm}\right)=\sqrt{{\left[\Delta \delta \left({}^{1}\text{H}\right)\right]}^{2}+0.2{\left[\Delta \delta \left({}^{15}\text{N}\right)\right]}^{2}}$$

Competition experiments between TDG-N and SUMO-1 for DNA binding was performed with an equimolar ratio of ^15^N-labeled TDG-N and a 25-mer double-stranded DNA containing a G:T mismatch at 20 μM. Unlabeled SUMO-1 was then added to a final concentration of 80 μM.

### Glycosylase activity on G:T/U mismatches

DNA nicking assays were performed as described in on 25 mer dsDNA containing either a central G:T or G:U mismatch, or a canonical G:C pair as a control. Briefly, oligonucleotides corresponding to the complementary strand were labeled on the primary amine modified 3'-end with the AlexaFluor^® ^488 dye (Invitrogen, Molecular Probes) and oligonucleotide annealing was performed as described in the previous section. TDG proteins were incubated at 0.5 μM final concentrations with dsDNA at 5 μM in 80 μl nicking buffer (25 mM Hepes.KOH pH 7.8, 1 mM EDTA, 1 mM DTT) at 37°C. 20 μl aliquots were withdrawn at different incubation times. DNA was precipitated in 70% ethanol solution containing 300 mM NaCl then incubated with 0.01 N NaOH for 30 min at 50°C. Oligonucleotides were separated by denaturing polyacrylamide gel electrophoresis and quantified using a GeneGenius bioimaging system (SynGene, Ozyme). The SUMO-1 effect on TDG glycosylase activity was investigated in presence of 2.5 and 5 μM of SUMO-1 under the same conditions as described above. Three independent replicates of glycosylase reactions were made for every time point in the kinetic studies. Absence of SUMO-1 glycosylase activity was confirmed with 5 μM SUMO-1 without TDG on G:T and G:U-containing substrates. Turnover rates are calculated as described [9]. Briefly, the turnover rate is the ratio of abasic DNA molecules produced per molecule of enzyme (pmol product/pmol TDG) as a function of time.

## Abbreviations

AP: apurinic/apyrimidic; APE1: A/P-Endonuclease 1; BER: base excision repair; CBP: Creb (cAMP-responsive element binding protein)-binding protein; Dnmt3a: DNA-methyltransferase 3a; dsDNA: double-stranded DNA; HAT, histone acetyltransferase; HSQC: heteronuclear single quantum coherence spectroscopy; MUG: mismatch-specific uracil-DNA glycosylase; SBM: SUMO-binding motif; SRC-1: steroid receptor coactivator 1; SUMO: small ubiquitin-like modifier; TDG: Thymine-DNA Glycosylase; TDG-CAT: TDG catalytic domain; TDG-RD: TDG regulatory domain; UDG: Uracil-DNA Glycosylase.

## Authors' contributions

CSM did the protein purifications, the NMR experiments, the activity assays, and wrote the manuscript. JMW assisted the NMR experiments. HL and SE did the protein activity measurements. AB supervised the project and wrote the manuscript. All authors have read and approved the final manuscript.

## Supplementary Material

Additional file 1 **Figure S1. **Titration of ^15^N-TDG by SUMO-1. (A) Comparison of ^1^H projections extracted from the ^15^N-^1^H HSQC spectrum of ^15^N-labeled TDG in presence of either 1 (black) or 10 equivalents (red) SUMO-1, ^15^N-TDG-N (blue) or ^15^N-TDG alone (green). Lines corresponding to G7 as a reference, the RD residue T68 or the C-terminal residue G344 are depicted. All peaks are normalized on the G7 signal extracted from the HSQC of ^15^N-TDG:SUMO-1 1:10 complex. (B) Graphical representation of the relative RD (upper panel) and C-terminal (lower panel) signal intensities for some TDG residues in presence of 1- or 10-fold excess SUMO-1. The signals are normalized by the peak integration of the residue G7 which is not affected by SUMO-1 interaction. Click here for file

Additional file 2 **Figure S2. Interactions of SUMO-1 with TDG.** Comparison of ^1^H projections of the ^15^N-^1^H HSQC spectra of ^15^N-SUMO-1 (black) and ^15^N-SUMO-1 at 33 μM with 100 μM TDG (blue). Resonances of the unfolded N-terminal residues of SUMO-1 are annotated. Click here for file

Additional file 3 **Figure S3**. ^15^N-^1^H HSQC spectra and circular dichroism spectra of wild-type TDG and different mutants. (A, B) ^15^N-^1^H HSQC spectra of ^15^N-labeled TDG wild type (black) and (A) TDG-E310Q at 100 μM (red) or (B) TDG-D133A at 45 μM (red) and TDG-D133A/E310Q at 50 μM (blue). (C) Comparison of circular dichroism spectra of wild-type TDG (blue), TDG-D133A (green), TDG-E310Q (orange) and TDG-D133A/E310Q (pink). The arrow indicates the difference of α-helix content for both TDG wild-type and TDG-E310Q on one hand, and both TDG-D133A and TDG-D133Q/E310Q on the other hand. (D) NMR samples of 300 μl ^15^N-labeled proteins (1) TDG wild type (1 μl) and (2) TDG-E310Q (3 μl), (3) TDG-D133A (5 μl) and (4) TDG-D133A/E310Q (5 μl) mutants obtained from *E. coli *cultures in M9 minimal medium. A higher molecular weight band is observed for the TDG-E310Q protein that could be due to TDG oxidation or contamination. This band is also detected for TDG wild-type to a lesser extent. Note, however, that the amount of total proteins loaded on the gel is also lower in lane 1. Click here for file

Additional file 4 **Figure S4**. Circular dichsoism spectra of wild-type TDG, TDG-E310Q and SUMO-1. Comparison of circular dichroism spectra of TDG-E310Q and wild-type TDG SUMO-1 equimolar complexes (pink) versus the sum of SUMO-1 and TDG proteins spectra (dark blue). Spectra of free SUMO-1 (green) and TDG proteins (light blue) are shown as references. Click here for file

Additional file 5 **Figure S5**. ^15^N-^1^H HSQC spectra of wild-type TDG, sumoylated TDG, TDG-E310Q and sumoylated TDG-E310Q in the presence or absence of SUMO-1. (A) Overlay of ^15^N-^1^H HSQC spectra of ^15^N-labeled TDG at 20 μM in the presence of 200 μM SUMO-1 (black), ^15^N-labeled sumoylated TDG at 100 μM (blue) and ^15^N-labeled sumoylated TDG at 20 μM in the presence of 200 μM SUMO-1 (red). Resonances of broadened C-terminal residues are annotated in italic characters and resonances of TDG-RD in bold characters. (B) Comparison of ^15^N-^1^H HSQC spectra of the ^15^N-labeled sumoylated (red) and unmodified (black) TDG-E310Q mutant at 100 μM. Resonances of ^15^N-labeled SUMO-1 N-terminal resonances are indicated by arrows. Click here for file

Additional file 6 **Figure S6**. ^15^N-^1^H HSQC spectra of wild-type TDG and TDG-N-terminus in presence of dsDNA with G:C pair, G:U or G:T mispair. Overlay of ^15^N-^1^H HSQC spectra of TDG alone at 20 μM (black) and in the presence of 50 μM of a 37-mer double-stranded DNA substrate (red) containing either a G:C pair (A), a G:U (B) or a G:T mismatch (C). The spectrum of the isolated N-terminus (TDG-N, residue 1 to 111) in the presence of DNA is represented in blue (A) as a reference. Click here for file
