# Supplementary figures and images for: SUMO-1 regulates the conformational dynamics of Thymine-DNA Glycosylase regulatory domain and competes with its DNA binding activity
Source: BMC Biochem. 2011 Feb 1;12:4. doi: 10.1186/1471-2091-12-4 (PMC3040724; doi:10.1186/1471-2091-12-4)

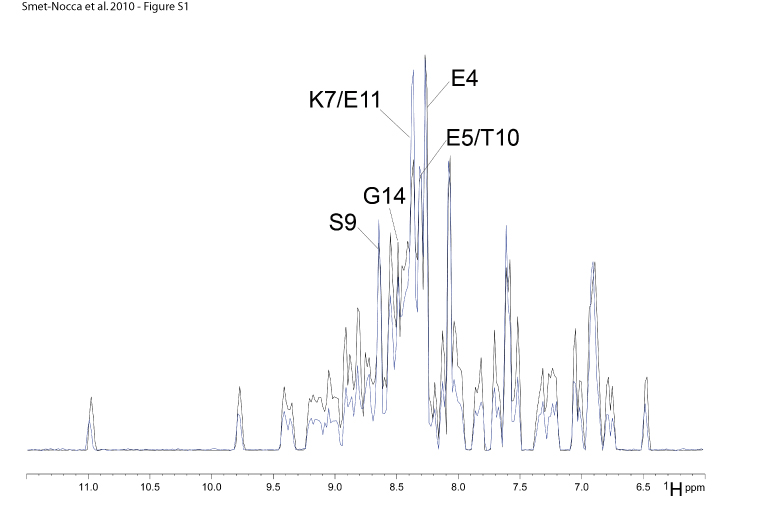

Supplement: Additional file 1 — Figure S1. Titration of 15N-TDG by SUMO-1. (A) Comparison of 1H projections extracted from the 15N-1H HSQC spectrum of 15N-labeled TDG in presence of either 1 (black) or 10 equivalents (red) SUMO-1, 15N-TDG-N (blue) or 15N-TDG alone (green). Lines corresponding to G7 as a reference, the RD residue T68 or the C-terminal residue G344 are depicted. All peaks are normalized on the G7 signal extracted from the HSQC of 15N-TDG:SUMO-1 1:10 complex. (B) Graphical representation of the relative RD (upper panel) and C-terminal (lower panel) signal intensities for some TDG residues in presence of 1- or 10-fold excess SUMO-1. The signals are normalized by the peak integration of the residue G7 which is not affected by SUMO-1 interaction. [file 1471-2091-12-4-S1.JPEG]

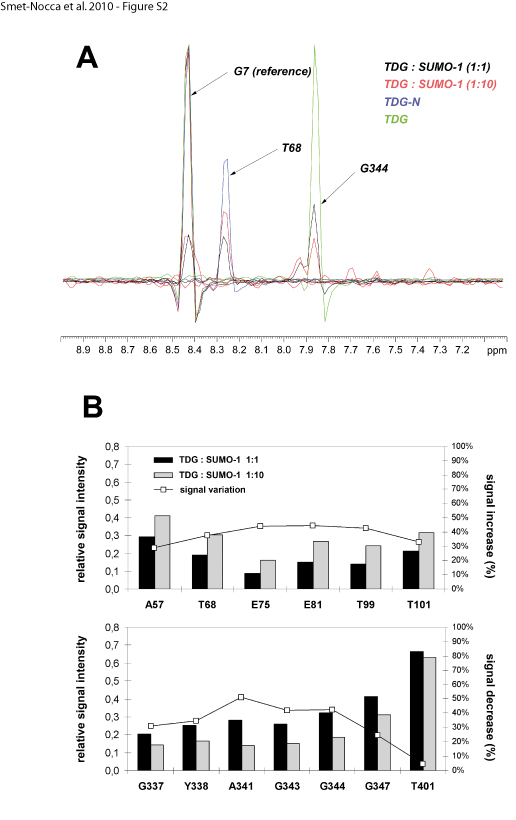

Supplement: Additional file 2 — Figure S2. Interactions of SUMO-1 with TDG. Comparison of 1H projections of the 15N-1H HSQC spectra of 15N-SUMO-1 (black) and 15N-SUMO-1 at 33 μM with 100 μM TDG (blue). Resonances of the unfolded N-terminal residues of SUMO-1 are annotated. [file 1471-2091-12-4-S2.JPEG]

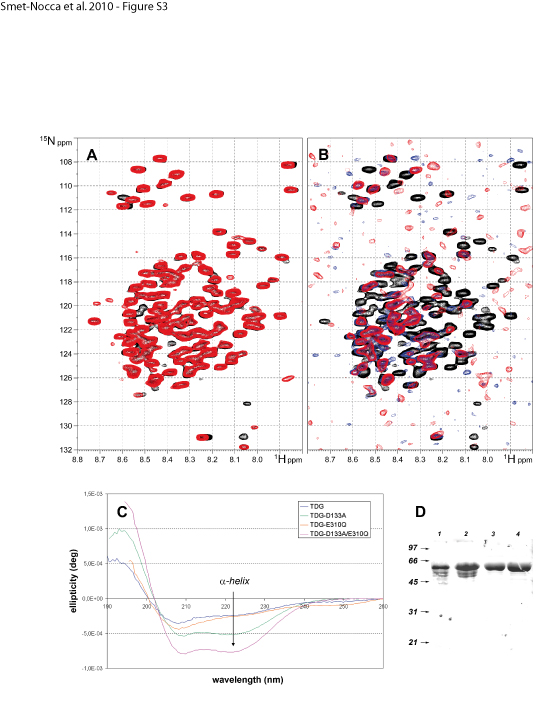

Supplement: Additional file 3 — Figure S3. 15N-1H HSQC spectra and circular dichroism spectra of wild-type TDG and different mutants. (A, B) 15N-1H HSQC spectra of 15N-labeled TDG wild type (black) and (A) TDG-E310Q at 100 μM (red) or (B) TDG-D133A at 45 μM (red) and TDG-D133A/E310Q at 50 μM (blue). (C) Comparison of circular dichroism spectra of wild-type TDG (blue), TDG-D133A (green), TDG-E310Q (orange) and TDG-D133A/E310Q (pink). The arrow indicates the difference of α-helix content for both TDG wild-type and TDG-E310Q on one hand, and both TDG-D133A and TDG-D133Q/E310Q on the other hand. (D) NMR samples of 300 μl 15N-labeled proteins (1) TDG wild type (1 μl) and (2) TDG-E310Q (3 μl), (3) TDG-D133A (5 μl) and (4) TDG-D133A/E310Q (5 μl) mutants obtained from E. coli cultures in M9 minimal medium. A higher molecular weight band is observed for the TDG-E310Q protein that could be due to TDG oxidation or contamination. This band is also detected for TDG wild-type to a lesser extent. Note, however, that the amount of total proteins loaded on the gel is also lower in lane 1. [file 1471-2091-12-4-S3.JPEG]

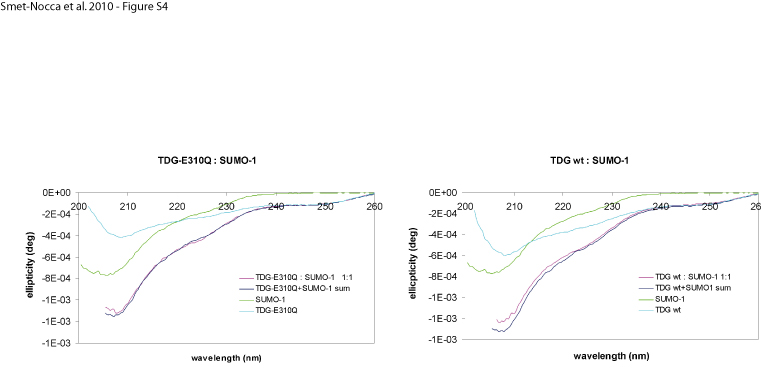

Supplement: Additional file 4 — Figure S4. Circular dichsoism spectra of wild-type TDG, TDG-E310Q and SUMO-1. Comparison of circular dichroism spectra of TDG-E310Q and wild-type TDG SUMO-1 equimolar complexes (pink) versus the sum of SUMO-1 and TDG proteins spectra (dark blue). Spectra of free SUMO-1 (green) and TDG proteins (light blue) are shown as references. [file 1471-2091-12-4-S4.JPEG]

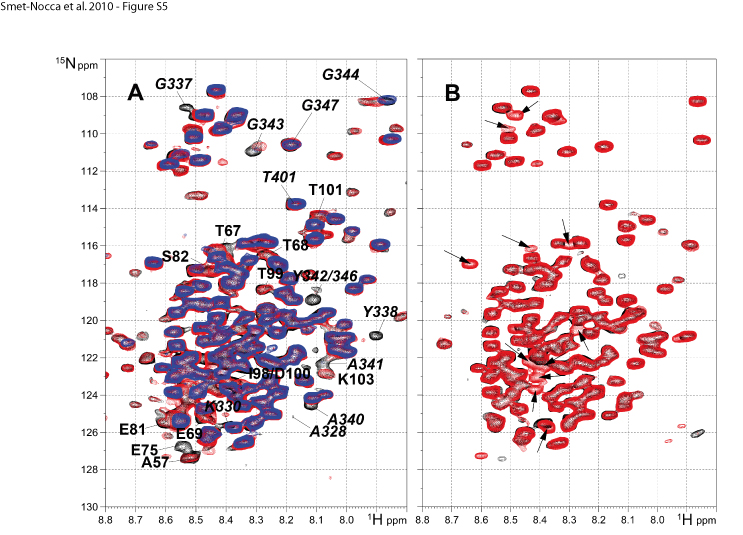

Supplement: Additional file 5 — Figure S5. 15N-1H HSQC spectra of wild-type TDG, sumoylated TDG, TDG-E310Q and sumoylated TDG-E310Q in the presence or absence of SUMO-1. (A) Overlay of 15N-1H HSQC spectra of 15N-labeled TDG at 20 μM in the presence of 200 μM SUMO-1 (black), 15N-labeled sumoylated TDG at 100 μM (blue) and 15N-labeled sumoylated TDG at 20 μM in the presence of 200 μM SUMO-1 (red). Resonances of broadened C-terminal residues are annotated in italic characters and resonances of TDG-RD in bold characters. (B) Comparison of 15N-1H HSQC spectra of the 15N-labeled sumoylated (red) and unmodified (black) TDG-E310Q mutant at 100 μM. Resonances of 15N-labeled SUMO-1 N-terminal resonances are indicated by arrows. [file 1471-2091-12-4-S5.JPEG]

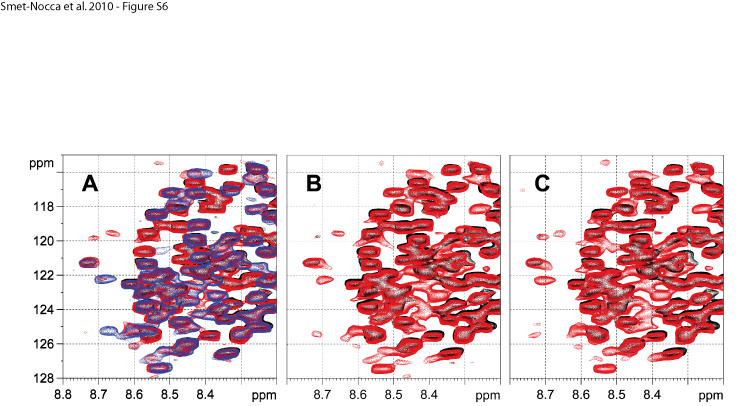

Supplement: Additional file 6 — Figure S6. 15N-1H HSQC spectra of wild-type TDG and TDG-N-terminus in presence of dsDNA with G:C pair, G:U or G:T mispair. Overlay of 15N-1H HSQC spectra of TDG alone at 20 μM (black) and in the presence of 50 μM of a 37-mer double-stranded DNA substrate (red) containing either a G:C pair (A), a G:U (B) or a G:T mismatch (C). The spectrum of the isolated N-terminus (TDG-N, residue 1 to 111) in the presence of DNA is represented in blue (A) as a reference. [file 1471-2091-12-4-S6.JPEG]
